# Characterization of MgCl_2_·6H_2_O-Based Eutectic/Expanded Perlite Composite Phase Change Material with Low Thermal Conductivity

**DOI:** 10.3390/ma11122369

**Published:** 2018-11-25

**Authors:** Chao Zhang, Zeyu Zhang, Rongda Ye, Xuenong Gao, Ziye Ling

**Affiliations:** 1 Key Laboratory of Enhanced Heat Transfer and Energy Conservation, the Ministry of Education, School of Chemistry and Chemical Engineering, South China University of Technology, Guangzhou 510640, China; hbjmzc@outlook.com (C.Z.); ye.rongda@mail.scut.edu.cn (R.Y.); cexngao@scut.edu.cn (X.G.); 2 School of Materials Science and Engineering, Northeastern University, Shenyang 110819, China; realfrankzhang@gmail.com; 3 Guangdong Engineering Technology Research Center of Efficient Heat Storage and Application, South China University of Technology, Guangzhou 510640, China

**Keywords:** phase change material, hydrated salt, CaCl_2_∙6H_2_O, melting point, MgCl_2_∙6H_2_O

## Abstract

The melting points of the phase change materials (PCMs) incorporated into the walls of buildings should be within the human thermal comfort temperature range. In this paper, 15 wt.% of MgCl_2_·6H_2_O was mixed with CaCl_2_·6H_2_O to obtain the eutectic with a melting point of 23.9 °C. SrCl_2_·6H_2_O suppresses the supecooling of the eutectic. The combination with expanded perlite (EP) via the impregnation method overcomes the phase separation and liquid leakage of the CaCl_2_∙6H_2_O-MgCl_2_∙6H_2_O mixture. The composite PCM is form-stable with the maximum loading mass fraction up to 50 wt.% and latent heat of 73.55 J/g. EP also significantly reduces the thermal conductivity of the CaCl_2_∙6H_2_O-MgCl_2_∙6H_2_O from 0.732 to 0.144 W/(m·K). The heating-cooling cycling test reveals that the composite PCM is thermally stable. The cheap eutectic salt hydrate, with little supercooling, no phase separation and liquid leakage, low thermal conductivity and good thermal reliability, show great potential as envelope materials to save energy consumption in buildings.

## 1. Introduction

A greater thermal mass and better thermal insulation of building envelopes provides improved thermal comfort, thus reducing the heating/cooling load. Phase change materials (PCMs) have large thermal mass because they can store and release a large amount of heat within a small temperature range during the phase change process between liquid and solid [1,2]. Therefore, incorporating PCMs with high heat capacity into building envelopes will definitely save energy consumptions in buildings [3,4].

The perfect PCM for building envelopes should be safe and cheap. They should also have high thermal storage density and a suitable phase change temperature within the human thermal comfort zone, which is between 22–26 °C. Compared to fatty acids or other organic PCMs [5], inorganic PCMs—such as salt hydrates, molten salts, etc.—offer the advantages of nonflammability and larger thermal storage density [6]. Hydrated salts are quite cheap due to rich resources in salt lakes [7,8]. Therefore, inorganic PCMs have recently gained intensive attention in building energy savings [9,10,11]. CaCl_2_∙6H_2_O is an inorganic hydrate with a melting point of 28 °C [12]. As reported by Li et al. [13], the melting point of CaCl_2_·6H_2_O can be regulated to 21–26 °C if it is mixed with MgCl_2_∙6H_2_O at certain ratios. As inorganic PCMs with a melting point of 22–26 °C are rarely reported, CaCl_2_∙6H_2_O-MgCl_2_∙6H_2_O eutectic is a great choice. However, even with all its advantages, the CaCl_2_·6H_2_O-MgCl_2_∙6H_2_O eutectic is still not yet commercially available for building envelopes.

First, the relatively high thermal conductivity gives the hydrated eutectic a poorer thermal insulation performance than the organic PCMs. Thus, the extra heat exchange between the building and the ambient will cause higher cooling/heating loads. Second, eutectics have inherent shortcomings such as supercooling and phase separation. More energy is consumed to extract the heat stored in the PCM if it is supercooled. Phase separation may cause the salt hydrate to stop functioning once its composition has changed. Unless the problems of high thermal conductivity, high supercooling degree and phase separation can be solved, the CaCl_2_·6H_2_O-MgCl_2_∙6H_2_O eutectic cannot be integrated into building envelopes for energy saving efforts.

Nucleating agents such as SrCl_2_∙6H_2_O have been found effective to reduce the supercooling degree of CaCl_2_∙6H_2_O and its eutectics [14]. Ways of alleviating the phase separation include adding thickening agents such as Carboxymethyl cellulos (CMC) and absorbing the PCMs into porous supporting materials such as expanded graphite [15,16,17], silica [18] or expanded perlite [19,20]. The porous matrix is a tank to hold the melted PCM that is in liquid state with the capillary force, so that the liquid leakage of the PCM can be avoided. In the meantime, the hydrated salts are constrained into macro or micro pores, which prevents the salts from separating from the hydrates. If the porous matrix has low thermal conductivity like silica or expanded perlite, the thermal conductivity of the salt hydrates can be reduced as well. Therefore, if CaCl_2_·6H_2_O-MgCl_2_∙6H_2_O is composited with SrCl_2_∙6H_2_O and a porous matrix with low thermal conductivity, the problems that hinder its application in building envelopes will be solved simultaneously.

However, the preparation of CaCl_2_·6H_2_O-MgCl_2_∙6H_2_O composite PCM has not yet been reported. In this work, we prepare a series of CaCl_2_∙6H_2_O-MgCl_2_∙6H_2_O eutectics with different compositions and different melting points. Then the mixture with the optimal melting point for use in building is selected. After that, the eutectic is composited with SrCl_2_∙6H_2_O and expanded perlite (EP) to reduce the supercooling degree, ease phase separation, and prevent liquid leakage. We study the microstructure, thermal properties and thermal reliability of the obtained composite PCM. The aim of this paper is to prepare a composite PCM with good thermo-physical properties for building envelopes.

## 2. Experiment

### 2.1. Material Preparation

#### 2.1.1. Preparation of the Eutectic

CaCl_2_ (purity: 96%, Tianjin Kemiou Chemical Reagent Co., Ltd., Tianjin, China) was dissolved in distilled water (molar ratio: 1:6) to prepare CaCl_2_·6H_2_O. Then the mixture was melted in a thermal bath at a temperature of 50 °C for 15 min. Finally, the CaCl_2_·6H_2_O was cooled down to solid form.

Different amounts of MgCl_2_·6H_2_O (purity: 98%, Tianjin Kemiou Chemical Reagent Co., Ltd., Tianjin, China) were mixed with the CaCl_2_∙6H_2_O to prepare the CaCl_2_∙6H_2_O-MgCl_2_∙6H_2_O eutectics. The mass fractions of MgCl_2_·6H_2_O varied from 10% to 25% with an interval of 5%. Each sample was heated to 50 °C and kept for 1 h until they were fully melted.

#### 2.1.2. Preparation of the Composite PCM

The CaCl_2_∙6H_2_O-MgCl_2_∙6H_2_O mixture with a suitable phase change temperature and relatively high latent heat was selected as the PCM to be composited with 2 wt.% SrCl_2_∙6H_2_O (purity: 99.5%, Tianjin Kemiou Chemical Reagent Co., Ltd., Tianjin, China) and EP (average diameter: 2–5 mm, Henan Huitong Co., Ltd., Zhengzhou, China) with mass fractions of 50 wt.%, 55 wt.%, 60 wt.%, and 65 wt.%. The CaCl_2_∙6H_2_O-MgCl_2_∙6H_2_O was impregnated into the EP at 50 °C and at a pressure of 88.1 kPa in vacuum.

### 2.2. Characterization

A differential scanning calorimeter (DSC, Q20, TA Instruments, New Castle, DE, USA) was used to analyze the phase change characteristics of the pristine eutectic, and the composite PCMs. The DSC tests were carried out under the atmosphere of nitrogen at a flow rate of 50 mL per minute. The testing temperature rose up from −5 to 50 °C at a rate of 3 °C/min.

Under the nitrogen atmosphere (flowrate: 100 mL/min), a thermoanalyzer (STA409PC, Netzsch, Waldkraiburg, Germany) tested the mass change of each sample during the heating process from 25 °C to 600 °C at a rate of 10 °C per minute. Thermal gravimetric analysis (TGA) characterizes the thermal stability of those materials.

The thermal conductivity of the materials in solid state were measured with a thermal constant analyzer (TPS 2500, Hot Disk, Gothenburg, Sweden). The composite PCM was compressed into two cylinders 40 mm in diameter and 10 mm in height. The sensor (type: 7577, 2.01 mm in diameter, Hot Disk, Gothenburg, Sweden) was sandwiched between the two cylinders. The thermal conductivity of the EP and eutectic were also measured, respectively.

**Figure 1:**
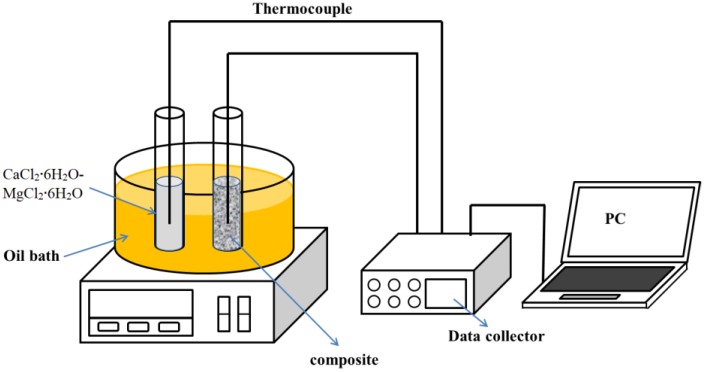
A schematic diagram of the experimental T-history set-up.

The supercooling degree of the PCMs was characterized by the cooling curves. The schematic diagram is shown in Figure 1. Two tubes that contained the eutectic and the composite PCM composites were put into a thermal bath in which the temperature was controlled by a thermal oil (Phenyl silicone oil, flash point: 315 °C, Clearco Products Co., Inc., Willow Grove, PA, USA). Two K-type thermocouples (NR-81530, accuracy: ±0.5 °C, HILA, Taipei, China) were placed at 20 mm from the bottom of each tube. First, the sample was heated to 50 °C and kept at this temperature for more than 10 min. Then, the thermal bath temperature was cooled down to −5 °C at a rate of 1 °C/min. After 10 min at −5 °C, the oil was heated to 50 °C again. The two thermocouples were connected to a data logger (34970A, Agilent, Santa Clara, CA, USA), which recorded the data of the temperature variations for each sample.

To determine whether the composite PCM was thermally reliable, the composite PCMs underwent 500 heating and cooling thermal cycles. In each cycle, the composite PCM was cycled between 50 °C and −5 °C via a temperature-controlled instrument (BPHJS-060A, Bluepard, Shanghai, China). In each stage of the thermal cycling, the composite PCM sample was maintained at the specified temperature for 30 min to ensure that the phase change completed. The results from SEM, Fourier transform infrared spectra, and DSC were compared before and after the thermal cycling.

The X-ray diffractometer (XRD, D8-ADVANCE, Bruker, Billerica, MA, USA, Cu Kα radiation λ = 1.5406 Å) collected the diffraction patterns of CaCl_2_∙6H_2_O, MgCl_2_∙6H_2_O, and the eutectics in the 2θ ranging from 5° to 80°. The FT-IR of the pristine salts and the composite PCMs whose wavenumber ranged from 4000–400 cm^−1^ were obtained with the spectrophotometer (Tensor 27, Bruker, Billerica, MA, USA) at room temperature. The microstructures of EP and the CaCl_2_∙6H_2_O-MgCl_2_∙6H_2_O/EP composite PCM were observed using a scanning electron microscope (SEM, Quanta, FEI, Hillsboro, OR, USA).

## 3. Results and Discussion

### 3.1. Determining the Suitable Mass Fraction of MgCl_2_∙6H_2_O

**Figure 2:**
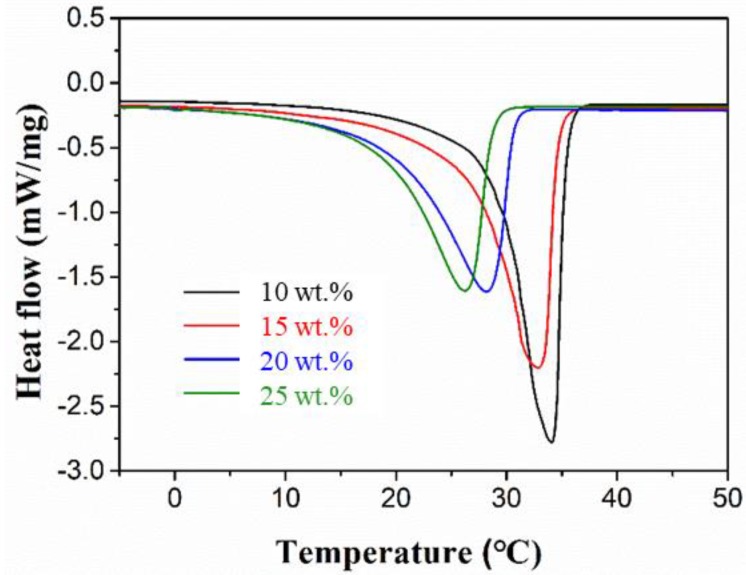
DSC curves of the CaCl_2_∙6H_2_O-MgCl_2_∙6H_2_O eutectic mixtures with different mass fractions of MgCl_2_∙6H_2_O.

Figure 2 shows the DSC curves of the CaCl_2_∙6H_2_O-MgCl_2_∙6H_2_O eutectic mixtures with different mass fractions of MgCl_2_·6H_2_O; the details are listed in Table 1. The increase in the mass fraction of MgCl_2_·6H_2_O leads to a decrease in the melting point and specific phase change enthalpy. Note that the suitable phase change temperature of the PCM applied into the walls of buildings has to be between 22 °C and 26 °C. Moreover, the peak temperature of the mixture containing 15% of MgCl_2_·6H_2_O is lower than that of the one containing 10%. Therefore, the mixture containing 15% of MgCl_2_·6H_2_O was selected as a suitable PCM for use in buildings, exhibiting a latent heat value of 151.9 kJ/kg.

**Figure 3:**
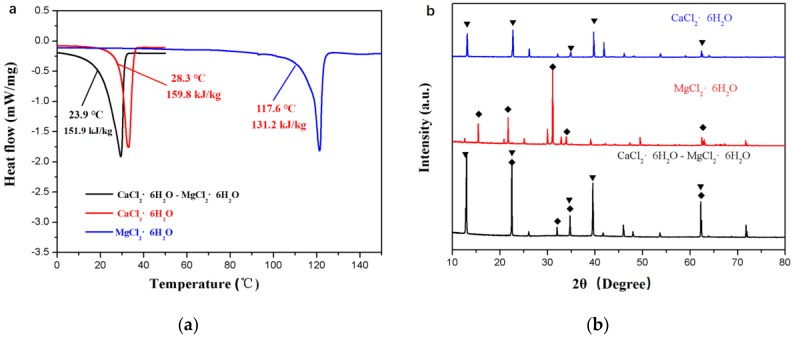
(**a**) DSC curves and (**b**) XRD patterns of the CaCl_2_∙6H_2_O sample, MgCl_2_∙6H_2_O, and CaCl_2_∙6H_2_O-MgCl_2_∙6H_2_O.

**Figure 4:**
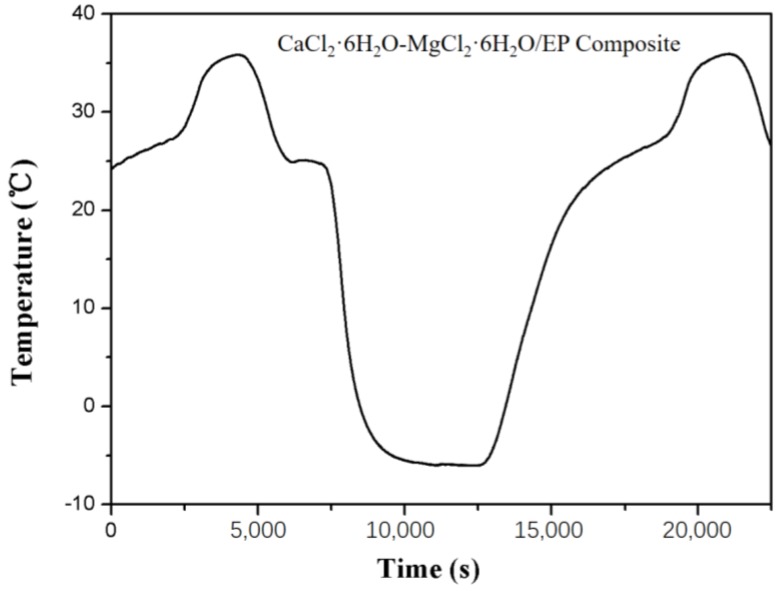
The cooling curve of the CaCl_2_∙6H_2_O-MgCl_2_∙6H_2_O mixture.

Furthermore, the DSC curves of the CaCl_2_∙6H_2_O sample, MgCl_2_∙6H_2_O, and the CaCl_2_∙6H_2_O-MgCl_2_∙6H_2_O PCM are illustrated in Figure 3a. The CaCl_2_∙6H_2_O sample has a melting point of 28.3 °C, slightly higher than the comfortable temperature range for human beings. Significantly, the addition of MgCl_2_∙6H_2_O with a phase change temperature of around 117 °C makes the obtained CaCl_2_∙6H_2_O-MgCl_2_∙6H_2_O mixture exhibit a melting point of 23.9 °C, lower than that of the CaCl_2_∙6H_2_O sample. Consequently, the CaCl_2_∙6H_2_O-MgCl_2_∙6H_2_O is more suitable for use in walls of buildings for energy conservation. Moreover, Figure 4b displays the XRD patterns of the CaCl_2_∙6H_2_O sample, MgCl_2_∙6H_2_O, and CaCl_2_∙6H_2_O-MgCl_2_∙6H_2_O PCM. It can be seen that the CaCl_2_∙6H_2_O-MgCl_2_∙6H_2_O PCM shows similar XRD pattern to that of the CaCl_2_∙6H_2_O sample; no diffraction peaks of MgCl_2_∙6H_2_O are observed, due to its low content in this mixture.

Before the mixture containing 15 wt.% of MgCl_2_∙6H_2_O is employed for practical applications, its supercooling degree needs to be evaluated. Consequently, its cooling curve has been measured by the T-history method. As displayed in Figure 4, no obvious supercooling is found for the CaCl_2_∙6H_2_O-MgCl_2_∙6H_2_O mixture, which is attributed to the addition of SrCl_2_∙6H_2_O as the nucleating agent. With the help of MgCl_2_·6H_2_O and SrCl_2_·6H_2_O, the suitable melting point, along with low supercooling degree, is achieved.

### 3.2. Characteristics of the Composite PCM

**Figure 5:**
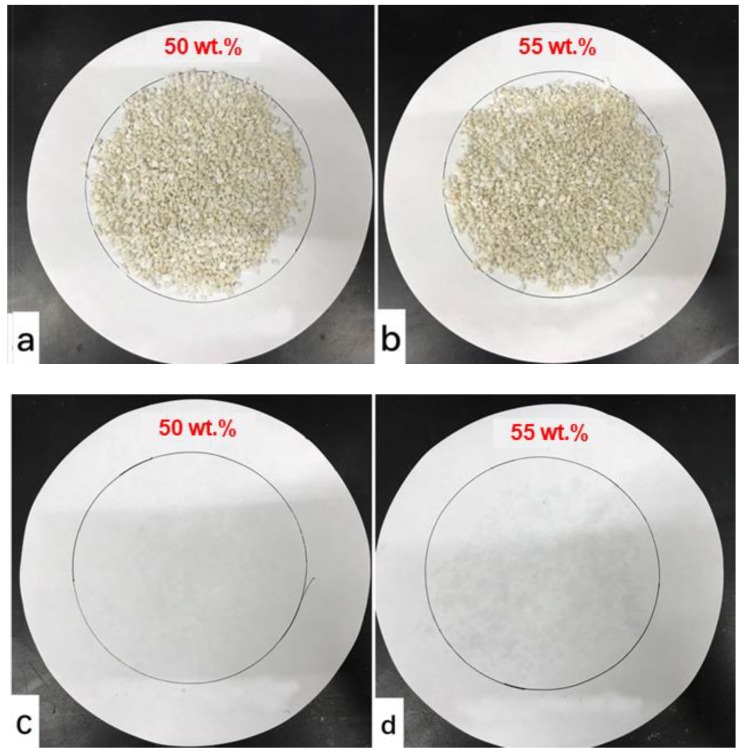
Photographs of the composite PCM containing 50% (**a**) and 55% (**b**) of the PCM, along with their corresponding filter paper.

The disadvantages of phase separation and liquid leakage for the eutectic mixture have been overcome by combining it with EP to prepare a composite PCM. In order to determine the adsorptive capacity of EP for the eutectic mixture, the liquid leakage tests have been conducted to the four composites containing different mass fractions of the eutectic. As shown in Figure 5, the composite containing 50% of the eutectic exhibits no liquid leakage, while some leakage is found on the filter of the one with PCM mass fraction of 55%. Therefore, the suitable mass fraction of the eutectic PCM adsorbed into EP is determined to be around 50%, and the corresponding sample is thus taken as the CaCl_2_∙6H_2_O-MgCl_2_∙6H_2_O/EP composite PCM.

**Figure 6:**
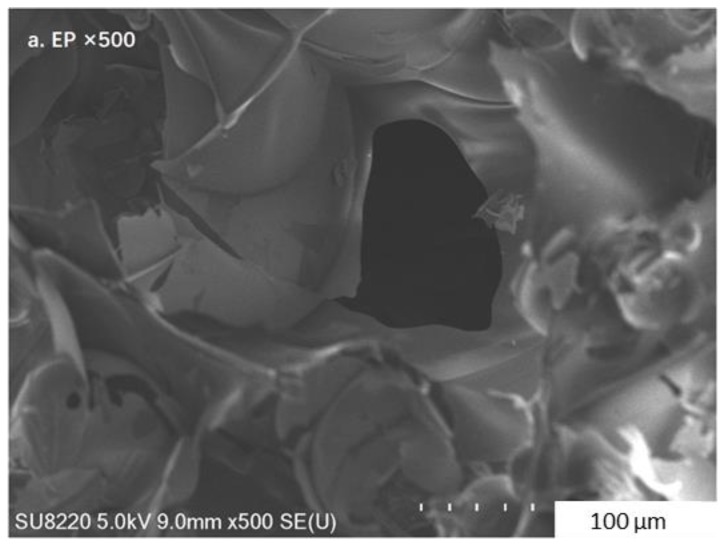

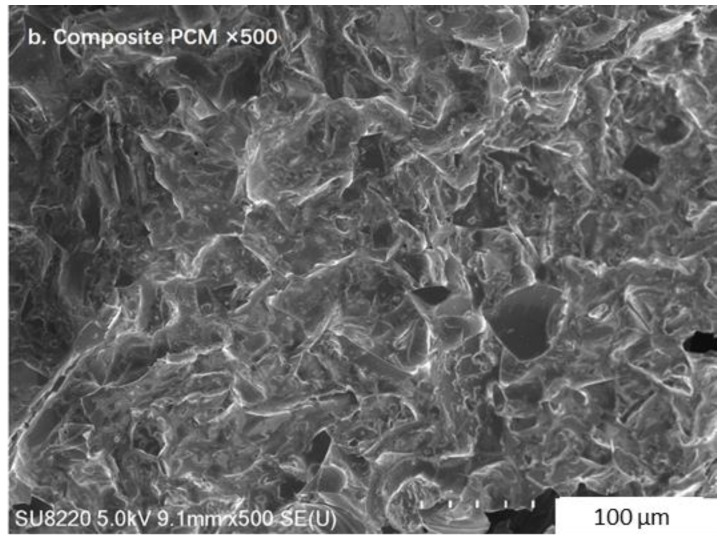
SEM images of EP (**a**) and the CaCl_2_∙6H_2_O-MgCl_2_∙6H_2_O/EP composite PCM (**b**).

The microstructure of the composite PCM containing 50% CaCl_2_∙6H_2_O-MgCl_2_∙6H_2_O has been observed by SEM, together with that of EP. As observed from Figure 6a, EP consists of thin sheets and pores, thereby possessing good adsorptive capacity. EP has been shown to be hydrophilic, thus having a good compatibility with hydrated salts. As shown in Figure 6b, the adsorbed hydrated salt is uniformly attached to the sheets of EP and filled into the pores.

**Figure 7:**
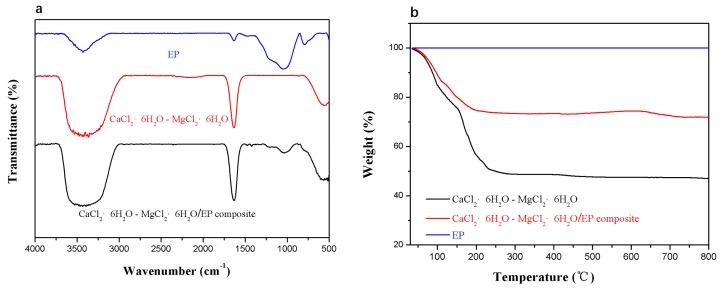
FT-IR spectra (**a**) and TG curves (**b**) of EP, the CaCl_2_∙6H_2_O-MgCl_2_∙6H_2_O eutectic mixture, and the CaCl_2_∙6H_2_O-MgCl_2_∙6H_2_O/EP composite PCM.

Figure 7a compares the FT-IR spectra of the EP, the pristine eutectic mixture, and the composite PCM. The FT-IR spectrum of the composite PCM combines the characteristics of EP and the eutectic mixture. The result suggests that EP physically interacts with the hydrated salt in the composite PCM. The thermal gravimetric analysis curves shows that the onset decomposition temperature of the composite PCM is slightly higher that of the pristine eutectic mixture in Figure 7b, which implies that the EP improves the stability of the eutectic mixture. The weight loss of the composite PCM is equivalent to the mass fraction of the eutectic mixture in it.

**Figure 8:**
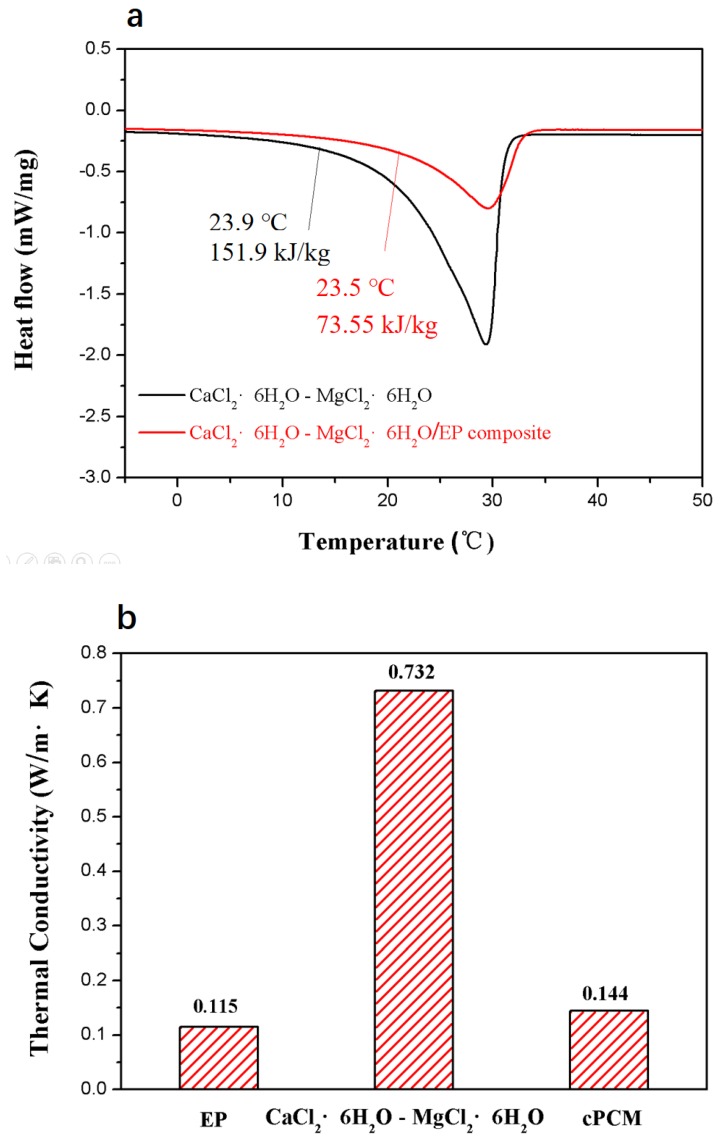
DSC data (**a**) and thermal conductivity values (**b**) of the materials.

Figure 8a shows the DSC curve of the composite PCM, along with that of the CaCl_2_∙6H_2_O-MgCl_2_∙6H_2_O mixture for comparison. The melting point of the composite PCM is 23.5 °C, very close to the CaCl_2_∙6H_2_O-MgCl_2_∙6H_2_O mixture, suggesting that the composite PCM can be applied into the walls of buildings for energy conversation. The specific phase change enthalpy of the composite PCM is 73.55 J/g, which agrees with the eutectic mass fraction in the composite PCM. Thanks to the low thermal conductivity of the EP, the thermal conductivity of the composite PCM is far lower than that of the eutectic, as shown in Figure 8b. Both the relatively high specific phase change enthalpy and the thermal conductivity guarantees that the composite PCM is suitable for use in energy-saving efforts for buildings.

### 3.3. Thermal Reliability of the Composite PCM

**Figure 9:**
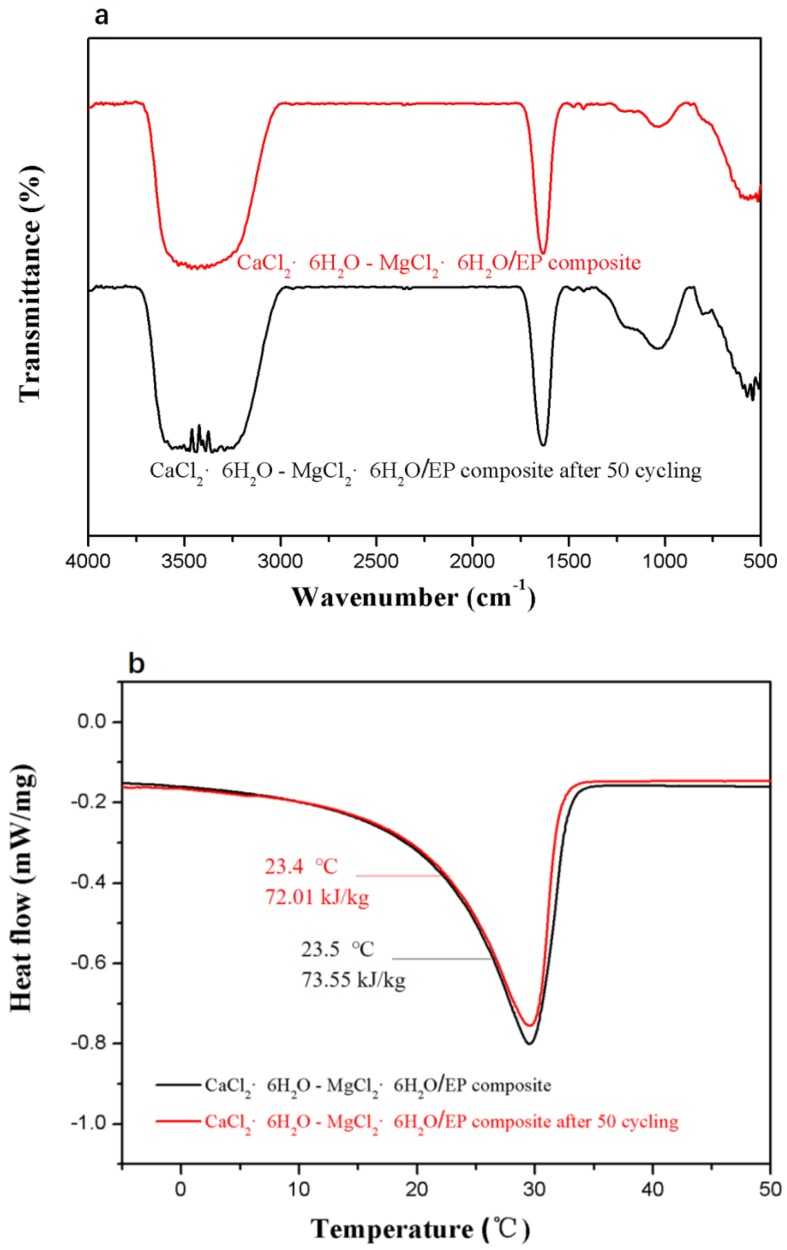
FT-IR spectra (**a**) and DSC curves (**b**) of the CaCl_2_∙6H_2_O-MgCl_2_∙6H_2_O/EP composite PCM that experiences (or does not) the heating-cooling cycling test.

Thermal reliability is critical to PCMs in practical applications. After experiencing 500 heating-cooling cycles, the physical properties of the composite PCM are compared with the sample without cycling. We find no obvious changes in the FT-IR spectra (Figure 9a) of the composite PCM before and after the heating-cooling cycling. These results suggest that composite PCM possess good structure stability. Moreover, the DSC curves of the composite PCM before and after the heating-cooling cycling almost overlaps, as shown in Figure 9b, indicating its excellent thermal reliability.

## 4. Conclusions

MgCl_2_∙6H_2_O has been mixed with CaCl_2_∙6H_2_O at different mass fractions to obtain a series of eutectic mixtures. It was found that the increase in mass fraction of MgCl_2_∙6H_2_O leads to reductions in melting point and latent heat. The mixture containing 15% of MgCl_2_·6H_2_O exhibits a melting point of 23.9 °C, latent heat of 151.9 J/g, and little supecooling, making it a suitable PCM for use in walls of buildings. The combination with expanded perlite has been employed for overcoming the phase separation and liquid leakage of the CaCl_2_∙6H_2_O-MgCl_2_∙6H_2_O mixture. The adsorptive capacity of EP for the mixture was determined to be around 50 wt.%, and the corresponding composite PCM shows a melting point of 23.5 °C and latent heat of 73.55 J/g. The thermal conductivity of the CaCl_2_∙6H_2_O-MgCl_2_∙6H_2_O/EP composite PCM is just slightly higher than that of EP, remarkably lower than that of the CaCl_2_∙6H_2_O-MgCl_2_∙6H_2_O mixture. The interaction between EP and the CaCl_2_∙6H_2_O-MgCl_2_∙6H_2_O mixture has been verified to be physical. The heating-cooling cycling test reveals that the composite PCM possesses good thermal reliability. Suitable phase change temperature, little supercooling, no phase separation and liquid leakage, low thermal conductivity, and good thermal reliability make the CaCl_2_∙6H_2_O-MgCl_2_∙6H_2_O/EP composite PCM show great promise for use in walls of buildings. The application of this composite PCM in buildings is under investigation.

## Figures and Tables

**Table 1:** Phase change characteristics of the CaCl_2_∙6H_2_O-MgCl_2_∙6H_2_O mixtures with different mass fractions of MgCl_2_∙6H_2_O.

MgCl_2_·6H_2_O (wt.%)	T_m-o_ (°C)	T_m-p_ (°C)	H_m_ (kJ/kg)
10	24.5	34.3	162.1
15	23.9	32.5	151.9
20	21.6	27.1	147.6
25	20.2	25.9	130.3
